# Antifungal activity of synthetic naphthoquinones against dermatophytes and opportunistic fungi: preliminary mechanism-of-action tests

**DOI:** 10.1186/1476-0711-13-26

**Published:** 2014-07-06

**Authors:** Maria do Perpetuo Socorro Borges Carriço Ferreira, Mariana Filomena do Carmo Cardoso, Fernando de Carvalho da Silva, Vitor Francisco Ferreira, Emerson Silva Lima, João Vicente Braga Souza

**Affiliations:** 1 Universidade Federal do Amazonas, Manaus, AM, Brazil; 2 Departamento de Química Orgânica, Universidade Federal Fluminense, Niterói, RJ, Brazil; 3 Instituto Nacional de Pesquisa.da Amazônia, Laboratório de Micologia, Manaus, AM, Brazil

**Keywords:** Naphthoquinones, Antifungal activity, Mechanism of action

## Abstract

This study evaluated the antifungal activities of synthetic naphthoquinones against opportunistic and dermatophytic fungi and their preliminary mechanisms of action. The minimum inhibitory concentrations (MICs) of four synthetic naphthoquinones for 89 microorganisms, including opportunistic yeast agents, dermatophytes and opportunistic filamentous fungi, were determined. The compound that exhibited the best activity was assessed for its action against the cell wall (sorbitol test), for interference associated with ergosterol interaction, for osmotic balance (K^+^ efflux) and for membrane leakage of substances that absorb at the wavelength of 260 nm. All tested naphthoquinones exhibited antifungal activity, and compound IVS320 (3a,10b-dihydro-1H-cyclopenta [b] naphtho [2,3-d] furan-5,10-dione)-dione) demonstrated the lowest MICs across the tested species. The MIC of IVS320 was particularly low for dermatophytes (values ranging from 5–28 μg/mL) and *Cryptococcus* spp. (3–5 μg/mL). In preliminary mechanism-of-action tests, IVS320 did not alter the fungal cell wall but did cause problems in terms of cell membrane permeability (efflux of K^+^ and leakage of substances that absorb at 260 nm). This last effect was unrelated to ergosterol interactions with the membrane.

## Introduction

The recent high incidence of fungal infections is due to the increased number of immunocompromised patients who are infected with HIV or have undergone organ transplantation or chemotherapy [1]. Although effective antifungal agents are currently available, side effects such as toxicity, drug interactions, inadequate pharmacokinetic properties and the development of resistance have been reported [2]. Therefore, new active entities that are safer, more potent and broad spectrum are highly desired as antifungal agents.

Among natural substances, naphthoquinones (found in bacteria, fungi, animals and plants) have attracted interest in recent years due to both their importance in vital biochemical processes and their several known biological activities, such as antitumor, antibacterial and antiviral [3]. Indeed, a series of studies have demonstrated that naphthoquinone derivatives exhibit antifungal activity [4,5]. Additionally, the antifungal activity of four synthesized compounds was reported against six species of *Candida* isolated from the oral cavities of patients with removable prostheses [6].

In this work, the antifungal activities of the compounds 2,2-dimethyl-2,3-dihydronaphtho [2,3-*b*] furan-4,9-dione, 2,2-dimethyl-2,3-dihydronaphtho [1,2-*b*] furan-4,5-dione, 3a,10b-dihydro-1H-cyclopenta [b] naphtho [2,3-d] furan-5,10-dione)-dione and 7,9a-dihydro-6bH-cyclopenta [b] naphtho [2,1-d] furan-5,6-dione were evaluated against 89 fungal cultures, including 29 opportunistic yeasts, 40 filamentous fungi and 20 opportunistic dermatophytes. The mechanism of action of the compound with the most effective antifungal activity was investigated using *Candida albicans* strain ATCC 36232.

## Methods

### Naphthoquinones

**Figure 1:**
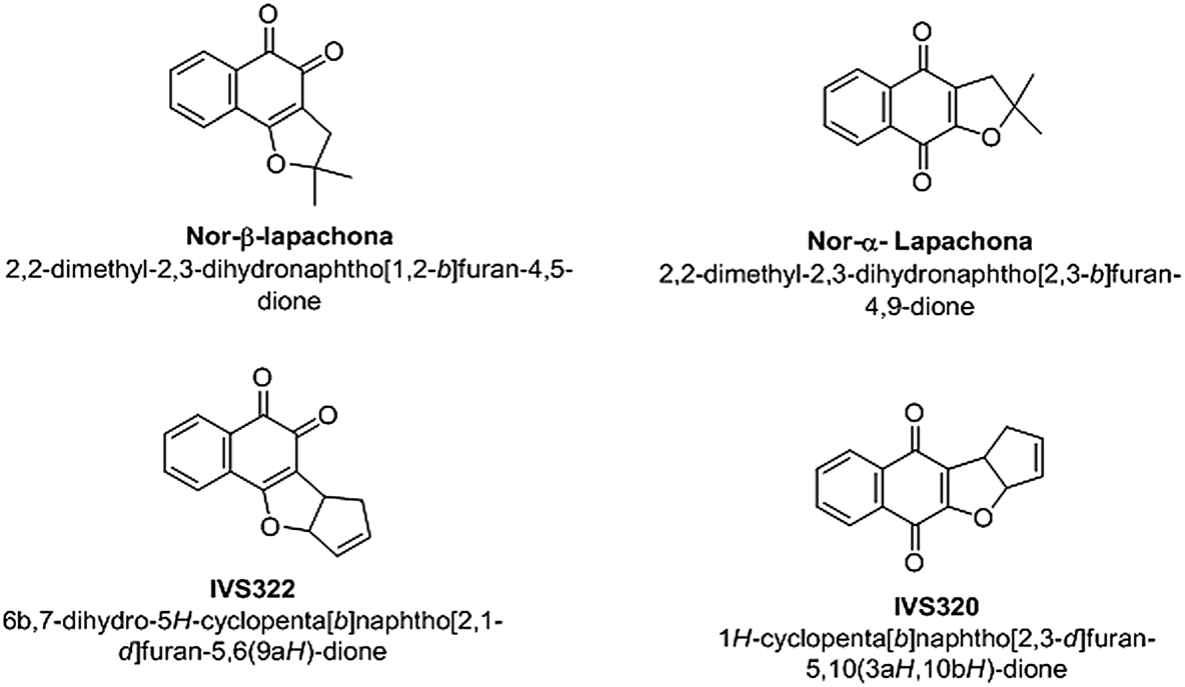
Structures of the naphthoquinones used in this study.

The compounds used in this study were the naphthoquinones IVS320 (3a,10b-dihydro-1H-cyclopenta [b] naphtho [2,3-d] furan-5,10-dione)-dione), IVS322 (7,9a-dihydro-6bH-cyclopenta [b] naphtho [2,1-d] furan-5,6-dione), nor-α-lapachone (2,2-dimethyl-2,3-dihydronaphtho [2,3-*b*] furan-4,9-dione) and nor-β-lapachone (2,2-dimethyl-2,3-dihydronaphtho [1,2-*b*] furan-4,5-dione) (Figure 1). These compounds were synthesized by the Developing Biologically Active Molecules and Methods for Organic Synthesis research group, Department of Organic Chemistry, Universidade Federal Fluminense-UFF, as described by Freire et al. [6]. A stock solution (2 mg/mL) of each compound was prepared and then diluted in RPMI 1640 (Sigma-Aldrich Co., St. Louis, MO, USA) to the required concentrations for the assays.

### Microorganisms

Eighty-nine cultures (Table 1) from the Collection of Microorganisms belonging to the National Institute of Amazon Research-INPA were used in this study. The cultures were preserved in mineral oil, and subcultures were maintained in Sabouraud medium to ensure purity and viability until the testing was performed. A culture of *C. albicans* (36232) from the American Type Culture Collection (ATCC) was used as a control for the susceptibility testing and mechanism-of-action studies.

**Table 1:** Microorganisms belonging to the INPA collection and from ATCC used in bioassays

**Group of organisms**	**Species (n)**	**Lines of code**
**Opportunistic yeasts**	*Candida albicans* (*n* = *1*)	ATCC 36232
	*Candida albicans* (*n* = *10*)	56/04; 377/06; 839/10; 113/01; 07/10; 143/05; 62/08; 100/04; 322/05; E2/09
	*Candida parapsilosis* (*n* = *09*)	U.1068; U.968/07; U.784/78; U.1047/08; 248/95; U1059/08; U864/07; U840; U1018/08
	*Cryptococcus neoformans* (*n* = *5*)	12/98; WM 148/10; WM626/10; WM628/10; WM629/10
	*Cryptococcus gattii* (*n* = *4*)	WM 161/10; WM178/10; WM179/10; WM 779/10
**Dermatophytes**	*Microsporum canis* (*n* = *10*)	93; Tcapt 178; D42; 794; 233; PV 78; Tcapt 209; 274; 88; Tcapt 215/03
	*Trichophyton rubrum* (*n* = *10*)	U80/99; U548; U656; U1185; U819; U1077; U855; Tp13; Tp269; Tcorp18
	*Trichophyton tonsurans* (*n* = *10*)	T. capt 300/08; U763/06; U763/06; U654/06; T corp 327/03; U1113/09; T corp 07; U68; U104; Tp 10 Tp42
	*Microsporum gypseum* (*n* = *10*)	PV 66/99; U1197/08; 680/07; 91/94; Tcorp 401/07; Tcorp 8/10; PV 56/99; Tcorp 494/08; T corp 282/02; Tcorp 467/08
**Opportunistic filamentous fungi**	*Aspergillus spp* (*n* = *10*)	U72; U841; U896; U918; U1124; Tp465; PV17; P640; PV672; P218
	*Fusarium spp* (*n* = *10*)	U1048/08; U37/10; U57/10; U998/08; U1028/08; U500/04; U1012/08; U557/04; U1128/09; U1118

### Antifungal activity assay

Minimum inhibitory concentration (MIC) assays were performed with the broth microdilution method, as described by the CLSI (Clinical and Laboratory Standards Institute) in documents M27-A2/CLSI [7] and M38-A [8]. Briefly, 100 μL of each evaluated compound diluted in RPMI 1640 broth was added to 96-well microplates, with the final concentrations of the compounds ranging from 100 to 0.2 μg/mL (naphthoquinones) and from 64 to 0.06 μg/mL (ketoconazole). Next, 100 μL of an inoculum containing 2.5 × 10^3^ cells/mL of opportunistic yeasts, 2.5 × 10^5^ cells/mL of dermatophytosis agents or 2.5 × 10^4^ cells/mL of opportunistic filamentous fungi was added to the microplate. The microdilution plates were incubated at room temperature (35°C) for 24 to 48 hours (opportunistic yeasts), 15 days (ringworm agents) or 3 days (opportunistic filamentous fungi). Visual readings were performed after 24 hours for the opportunistic yeasts, after 48 hours for the opportunistic filamentous fungi and after 120 hours for the dermatophytes. The MIC was defined as the lowest concentration of the compound causing 50% inhibition of microorganism growth compared to the control without inhibitors.

### Mechanism-of-action assays

The antifungal mechanism of action of naphthoquinone IVS320 (3a,10b-dihydro-1H-cyclopenta [b] naphtho [2,3-d] furan-5,10-dione)-dione) was evaluated using the yeast *C. albicans* strain ATCC 36232 as a model. The influence of IVS320 on the cell wall (sorbitol protection assay) and the effect of ergosterol on the cell membrane (ergosterol effect assay, K^+^ efflux assay and leakage of substances absorbing at 260 nm) were evaluated.

### Sorbitol protection assay

We determined the MIC of IVS320 against *C. albicans* (ATCC 36232) following CLSI guidelines (from 100 to 0.20 μg/mL) in the presence and absence of 0.8 M sorbitol (Sigma-Aldrich), which acts as an osmotic support. The MICs were determined after 24 hours of incubation at 35°C [9,10].

### Ergosterol effect assay

The MIC of IVS320 for *C. albicans* (ATCC 36232) was determined following CLSI guidelines in the presence and absence of different ergosterol concentrations (Sigma-Aldrich) (200–800 μg/mL), as previously described [10,11]. Amphotericin B was used as a control in this assay. The MICs were determined after 24 hours of incubation at 35°C.

### Potassium efflux assay

*C. albicans* (ATCC 36232) was grown for 18 hours at 35°C in RPMI medium. The cells were washed and resuspended to a concentration of 2.5 × 10^3^ cells/mL in deionized water, and 1 mL of this suspension was incubated with IVS320 at the MIC concentration (50 μg/mL) in test tubes at 35°C for various time periods [12]. *C. albicans* incubated with deionized water only was used as a control. After centrifugation, the amount of K^+^ released into the supernatant was measured using an atomic absorption spectrophotometer (AA Spectrophotometer 2380, Perkin-Elmer).

### Test for leakage of substances absorbing at 260 nm

*C. albicans* (ATCC 36232) was grown with shaking at 35°C until the early stationary phase (18 hours of growth) in RPMI. After incubation, *C. albicans* cells were washed and resuspended in MOPS buffer (0.16 M, pH 7.0). Microtubes (final volume 500 μL) containing an inoculum of 5 × 10^4^ cells/mL and 1× or 4× MIC concentrations of IVS320 were incubated for 6 hours. After 1, 2, 4 or 6 hours of incubation, the microtubes were centrifuged (5 min at 3,000 rpm), and the absorbance of the supernatants (100 μL) was measured at 260 nm (Gene Quant DNA/RNA Eppendorf). In this assay, the absorbance due to leakage from cells treated with HClO_4_ (1.2 M, 100°C, 30 min) was considered 100% [10,13].

## Results

### Antifungal activity of new naphthoquinones

To investigate the antifungal potential of synthetic naphthoquinones, the MIC of each compound was determined against 89 fungal cultures, including opportunistic yeasts, dermatophytes and opportunistic filamentous fungi (Table 1). The compound designated IVS320 (3a,10b-dihydro-1H-cyclopenta [b] naphtho [2,3-d] furan-5,10-dione)-dione) presented the lowest MICs for all the tested cultures and was particularly active against dermatophyte fungi and yeasts from the genus *Cryptococcus* (Table 2). Regarding dermatophyte fungi, the mean MIC ranged from 5–28 μg/mL; against *Cryptococcus* spp., the mean MIC ranged from 3–5 μg/mL.

**Table 2:** **Minimum inhibitory concentrations** (**MICs**) **of new naphthoquinones against different fungal species**

**Group microorganism**	**Culture (n** **=** **89)**	**Nor-alfa**		**Nor-beta**		**IVS320**		**IVS322**		**ketoconazole**	
		**Minimum Inhibitory Concentration (MIC) μg/mL**									
		**GM**	**Range**	**GM**	**Range**	**GM**	**Range**	**GM**	**Range**	**GM**	**Range**
**Yeasts opportunistic**	*C. albicans* (n = 10)	66	6.3 – 100	86	6.3 – 100	35	3.1-50	78	6,3-100	1	0,125-2
	*C. parapsilosis* (n = 10)	50	-	75	25-100	25	13-50	86	25-100	0,06	-
	*C. neoformans* (n = 05)	15	12.5-50	100	100	5	1,56-6,25	56	25-50	-	-
	*C. gattii* (n = 04)	25	12.5-50	100	100	3	1,56-6.25	44	25-50	-	-
**Dermatophytes**	*T. rubrum* (n = 10)	16	6.3-25	19	6.3-100	5	3,3-12,5	10	6,3-25	29	2-64
	*M. canis* (n = 10)	43	25-50	30	12,5-100	8	3,1-12,5	24	3,1-100	5	1-16
	*M. gypseum* (n = 10)	78	50-100	67	25-100	28	12,5-50	122	50-200	5	2-8
	*T. tonsurans* (n = 10)	35	12,5-50	34	6.3-200	8	3,11-25	38	25-50	1	0,5-4
**Filamentous opportunistic**	*Fusarium* sp. (n = 10)	117	50-200	67	25-100	36	25-50	103	50-200	6	1-16
	*Aspergillus* sp. (n = 10)	90	50-100	75	50-100	33	6,3-50	85	50-100	32	4-64

*GM* = geometric mean; − test not performed, n = number of different isolates.

The geometric mean and range for each compound against each group of fungal species are reported.

### Investigation of the mechanism of action of IVS320 against *C. albicans* ATCC 36232

#### Action on the cell wall

A sorbitol protection assay was conducted to determine the influence of IVS320 on the integrity of the fungal cell wall. In this assay, MIC determinations for IVS320 against *C. albicans* ATCC 36232 were carried out in parallel in the presence and absence of sorbitol (0.8 M), which is an osmotic protectant used for the stabilization of fungal protoplasts. If a compound negatively interferes with the fungal cell wall, it will shift the MIC to a higher value in the presence of osmotic support [9]. The MIC of IVS320 did not change in the presence of sorbitol (50 μg/mL) after 72 hours of incubation, which suggests that IVS320 does not act by inhibiting the mechanisms that control cell wall synthesis.

#### Action on ergosterol

**Figure 2:**
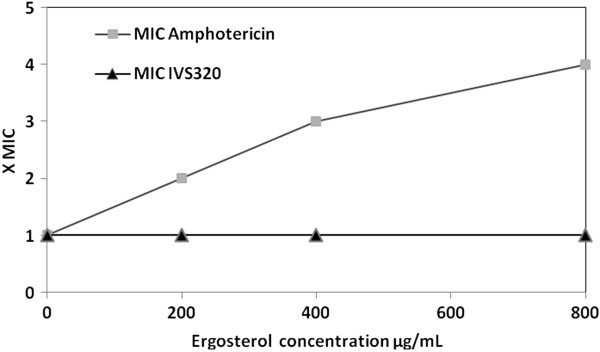
**Effects of exogenous ergosterol (200–800 μg/mL) on the MIC of IVS320 and amphotericin B against *C. albicans *ATCC 36232.** On the y-axis: 1 = 1× MIC, 2 = 2× MIC, 3 = 3× MIC, 4 = 4× MIC.

An ergosterol assay was used to determine whether IVS320 induces changes in the fungal membrane by interacting with ergosterol. This assay has been shown to identify compounds that bind ergosterol in fungal membranes and is based on the addition of exogenous ergosterol. A compound that has an affinity for ergosterol rapidly forms complexes with free ergosterol, thus preventing interactions with ergosterol in the fungal membrane; as a result, the MIC of the tested compound increases. The results showed that the MIC of IVS320 against *C. albicans* ATCC 36232 did not change in the presence of different concentrations (200 to 800 μg/mL) of exogenous ergosterol (Figure 2), suggesting that IVS320 does not act by interacting with ergosterol. In contrast, there was a 4× MIC increase with amphotericin B, which is known to have a strong affinity for ergosterol and served as the positive control in this assay [14].

#### Action on potassium efflux

**Figure 3:**
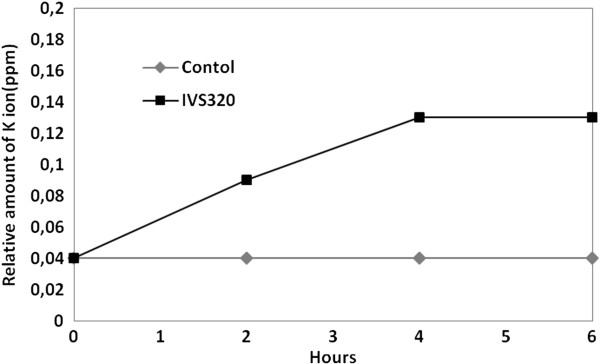
**Effect of IVS320 on K**^**+ **^**efflux from *C. albicans *** **ATCC 36232.**

A potassium efflux test was conducted to evaluate whether compound IVS320 interferes with the osmotic balance of *C. albicans* (ATCC 36232). This assay is based on the fact that the cell membrane is responsible for maintaining intracellular K+, so an increase in extracellular K + indicates cell membrane disruption. As shown in Figure 3, the efflux of K^+^ from *C. albicans* (ATCC 36232) cells into the extracellular medium increased with IVS320 treatment, whereas the extracellular K^+^ concentration in the control culture remained constant during incubation. The time course of K^+^ efflux into the culture supernatant indicated that the permeability of the fungal membrane increased up to 4 hours.

#### Leakage of substances absorbing at 260 nm

**Figure 4:**
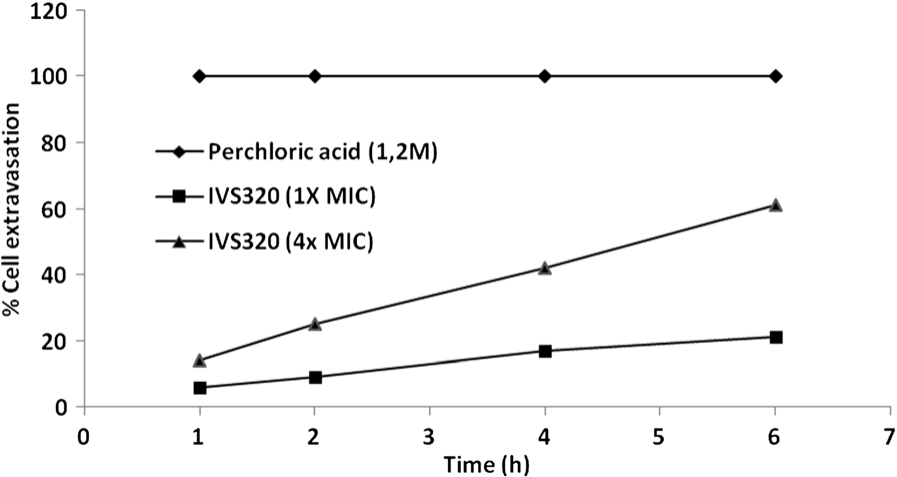
**Leakage of substances absorbing at 260 nm from *C. albicans *** **ATCC 36232 incubated (1–6 hours) with 1× and 4× MICs for IVS320.**

The interference of IVS320 with the membrane of *C. albicans* ATCC 36232 was determined by evaluating the leakage of substances that absorb at 260 nm from the membrane. Membrane rupture causes the release/leakage of intracellular components from the fungal cell, which can then be measured. Nucleotides, which exhibit a strong absorbance at 260 nm, are among the components that can be monitored to detect leakage. IVS320 (1× MIC and 4× MIC) was added to cell suspensions of *C. albicans*, and the samples were examined after various time intervals (1, 2, 4 and 6 hours). The results showed that 1× IVS320 MIC caused increases of 6, 9, 17 and 21% at 1, 2, 4 and 6 hours, respectively, compared to the perchloric acid control, which is considered to produce 100% cell leakage (Figure 4). Furthermore, the 4× MIC treatment caused a 2.6× increase in nucleotide leakage, resulting in 14, 25, 42 and 61% increases in leakage at 1, 2, 4 and 6 hours, respectively, compared to the perchloric acid control.

## Discussion

The 1,4-naphthoquinone structure is common in various natural products and clinically used drugs that are associated with antifungal activity. Previous studies have demonstrated the potential of this class of compounds. Importantly, the work of Sassaki et al. [15] evaluated the antifungal activities of 1,4-naphthoquinone derivatives and obtained MICs of 8 μg/mL and 16 μg/mL against cultures of *Candida albicans* and *Candida parapsilosis*, respectively. Sheng et al. 2011 [5] studied structural changes and the structure/activity of 1,4-naphthoquinone, showing that the introduction of side chains containing sulfur, oxygen or nitrogen atoms at position C2 and/or C3 led to an increase in antifungal activity. These researchers also evaluated the activity of 17 compounds with different side chains and found three compounds that showed broad antifungal activity. One compound, [2-chloro-3-((4-hydroxyphenyl) amino) naphthalene-1,4-dione], presented MICs of 3.12 μg/mL, 1.56 μg/mL and 0.78 μg/mL against *Aspergillus fumigatus*, *C. albicans* and *C. neoformans*, respectively. The antifungal potential of 1,4-naphthoquinone derivatives was also confirmed by Yamashita et al. [16] and Kategaonkar [17].

In the present work, all tested naphthoquinones exhibited antifungal activity. These results corroborate those of Freire et al. [6], who observed the inhibitory activity of these compounds against *C. albicans*, particularly Nor-α. Nonetheless, these findings require confirmation across a large number of *C. albicans* isolates as well as other fungal classes. The results of the present study clearly demonstrate the potential of these antifungals against a large number of different isolates of pathogenic species.

IVS320 exhibited the lowest MIC against dermatophytes and *Cryptococcus* spp. These results are important given that this is the first study evaluating the antifungal action of this compound against these agents. The importance of new drugs for the treatment of dermatophytosis is highlighted by the fact that this disease affects approximately 40% of the world population, that only a small number of drugs are currently available for treatment, that azoles and allylamines have adverse gastrointestinal effects and that there is a high frequency of recurrence [18,19]. Amphotericin B and flucytosine are widely used for the treatment of cryptococcosis; however, the toxicity of both of these therapeutics is well described, and therapeutic failure is often observed [20]. Due to the robust results for IVS320, this naphthoquinone was selected for assessing its antifungal mechanism of action. A strain of *Candida albicans* (ATCC 36232) was selected due to the importance of this species in the epidemiology of fungal infections, and the use of an ATCC microorganism will facilitate reproduction of the work reported here [21].

IVS320 did not alter the structure of the fungal cell wall but did change the permeability of the cell membrane (efflux of K^+^ and leakage of substances that absorb 260 nm), which was not related to binding with ergosterol. These results are similar to those obtained by Emadi et al. [22], who evaluated the possible mechanism of action of bis-naphthoquinones and concluded that these compounds cause membrane depolarization.

Accordingly, the toxicity of IVS320 should be extensively evaluated against human cells, even though studies by Freire et al. [6] showed that this compound exhibited no significant hemolytic activity against mouse erythrocytes and no cytotoxicity against human fibroblasts (NIH 3 T3) at concentrations between 12.5 and 50 μg/mL.

The results of the present study are relevant because they will motivate new in vivo research focusing on the development of new antifungal agents as alternatives for the treatment of ringworm and opportunistic mycoses.

## Competing interests

The authors declare that they have no competing interests.

## Authors’ contributions

JVBS: Study supervision, study concept and design, critical revision of the manuscript. VFF and ESL: Drafting of the manuscript, critical revision of the manuscript for important intellectual content. MPSBCF: Carried out the microbial assays, analysed the data and wrote the article. MFCC and FCS: Carried out drug synthesis. All authors read and approved the final manuscript.
